# P-1781. Development of an Inpatient Pharmacist-Driven Penicillin Allergy De-Labeling Protocol

**DOI:** 10.1093/ofid/ofae631.1944

**Published:** 2025-01-29

**Authors:** Brandon Anderson, Emily Herstine, Krista Gens

**Affiliations:** Abbott Northwestern Hospital, Minneapolis, Minnesota; Abbott Northwestern Hospital, Minneapolis, Minnesota; Abbott Northwestern Hospital, Minneapolis, Minnesota

## Abstract

**Background:**

Up to 10% of the US population reports a penicillin allergy. However, when further tested, over 95% of these patients could tolerate a penicillin. Unnecessary avoidance of penicillins results in more broad antibiotic treatment, leading to several worsened patient outcome, including increases in resistance, *C. difficile* rates, mortality, cost, and more.

Example Inpatient Penicillin Allergy De-Labeling Protocol

**Figure:**
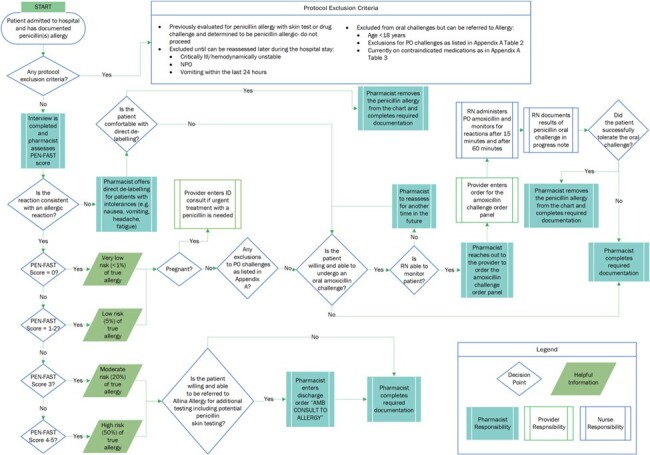

Visual representation of an example penicillin allergy de-labeling protocol

**Methods:**

Key penicillin allergy de-labeling protocol development steps included assessment of local penicillin allergy prevalence, identification of key stakeholder groups, effectively communicating why de-labeling is important, and incorporating feedback from each stakeholder group into protocol language. Operational considerations included creation of standardized order sets and documentation tools, as well as defining scope and prioritization of patients by determining who is most likely to benefit from de-labeling. The last step was development of educational materials for pharmacists and nurses to effectively use the protocol, as well as patient education materials.

**Figure:**
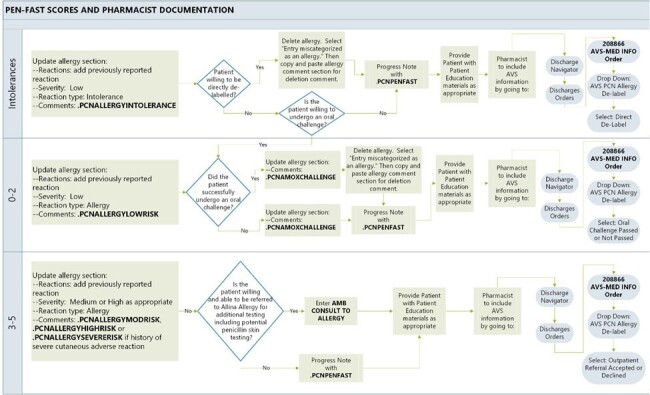

Flowsheet outlining documentation requirements, including standardized allergy comments, progress note templates, and after visit summaries

**Results:**

The Allina penicillin allergy de-labeling protocol was developed through collaborative efforts of pharmacy, infectious disease, allergy, and nursing. Within the protocol, patients with penicillin allergies listed in their charts are interviewed by a pharmacist, who calculates a PEN-FAST risk score. Patients with reactions that are consistent with intolerance rather than allergy may have their allergy removed without further testing. If reactions are consistent with allergy, but their PEN-FAST risk score is low (0-2), patients are eligible to complete an oral amoxicillin challenge. If PEN-FAST risk score is moderate to high (3-5), patients are eligible for referral to outpatient allergy for further testing.

De-Labeling Protocol Development Process

**Figure:**
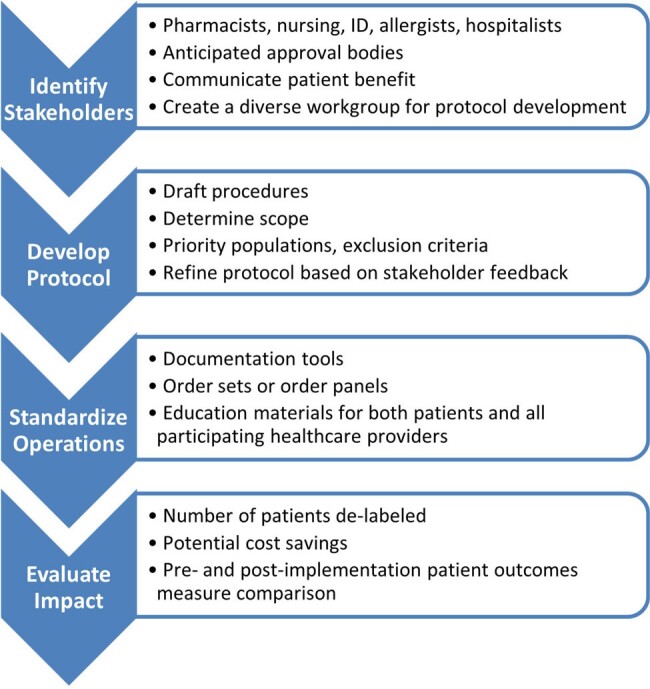

Example roadmap that can be used to develop a penicillin allergy de-labeling protocol in nearly any healthcare setting

**Conclusion:**

The penicillin allergy de-labeling protocol was approved system-wide through pharmacy and nursing committees and will soon be rolled out to individual sites. This example may serve as a roadmap for development of penicillin de-labeling programs in other health systems and could be adapted as needed to work effectively in many healthcare settings.

**Disclosures:**

**All Authors**: No reported disclosures

